# Association of *LOXL1* Gene Polymorphisms with Exfoliation Glaucoma Patients

**Published:** 2019-10

**Authors:** Elham TAGHAVI, Ramin DANESHVAR, Zahra NOORMOHAMMADI, Seyed Mohammad-Hossein MODARRESI, Mohammad Reza SEDAGHAT

**Affiliations:** 1. Department of Biology, Science and Research Branch, Islamic Azad University, Tehran, Iran; 2. Eye Research Center, Mashhad University of Medical Sciences, Mashhad, Iran; 3. Genetic Research Center, Mashhad University of Medical Sciences, Mashhad, Iran; 4. Department of Medical Genetics, School of Medicine, Tehran University of Medical Sciences, Tehran, Iran

**Keywords:** Pseudoexfoliation syndrome, *LOXL1* mRNA expression, Variants, Glaucoma

## Abstract

**Background:**

Pseudoexfoliation syndrome (XFS) is pathogenetically related to exfoliative glaucoma (XFG), which is the most common type of secondary glaucoma. We aimed to investigate the relationship between *LOXL1* SNPs (rs1048661, rs3825942) and XFS and/or XFG in a cohort of Iranian subjects.

**Methods:**

This cross-sectional study investigated possible association between LOXL1 gene polymorphisms and exfoliative glaucoma in Northeastern part of Iran between May 2014 and May 2015. Sixty unrelated XFS/XFG patients, as well as 40 control subjects, were studied by direct sequencing. In fifteen senile cataract patients without glaucoma and fifteen patients with coexisting XFG and cataract, capsulorhexis specimen of the anterior lens capsule was used to evaluate *LOXL1* gene transcripts by Real-Time PCR technique. We analyzed the results for allele frequencies and haplotype association and investigated the relative gene expression.

**Results:**

Significant associations between the rs382594 SNP and XFG and between rs1048661 SNP and XFG were observed (*P*<0.05 for both). The frequency of the G allele in the exonic SNP (rs1048661) appeared to be higher in XFS or XFG patients compared to control subjects (*P*= 0.0497). Moreover, in the rs3825942 SNP, the G allele was more frequent in XFS/XFG patients compared to control subjects (*P*=0.0016). The highest cumulative frequency was for the GG haplotype. GG haplotype was associated with increased risk of XFG compared to the rs1048661 G/T and rs3825942 G/A haplotypes. *LOXL1* mRNA expression was not statistically significantly different between XFS/XFG and control subjects.

**Conclusion:**

We reported the *LOXL1* gene polymorphism in an Iranian XFS/XFG cohort. Similar to many other ethnic groups and geographic regions, our results confirmed an association between *LOXL1* gene variants and XFG in Iran.

## Introduction

Exfoliation syndrome (XFS) is an age-related disease of the extracellular matrix (ECM), characterized by gradual accumulation of abnormal fibrillary material in various ocular and extraocular tissues. Lindberg described the condition for the first time in 1917 (1). This syndrome is the most common cause of secondary open-angle glaucoma and is associated with increased prevalence of cataract and complications during cataract surgery (2).

Glaucoma is a degenerative optic neuropathy caused by retinal ganglion cell apoptosis, with accompanying excavated optic disc cupping and characteristic visual field defect. In XFS, the exfoliative materials block the trabecular outflow pathway, which in turn cause a rise in intraocular pressure (3, 4). XFG accounted for up to 50% of open-angle glaucoma (5, 6). Despite many theories, the exact pathophysiological mechanism of glaucoma is still unknown. Some studies suggested XFS as complex disease caused by interaction between environmental factors and the genetic susceptibility of patients (7, 8). A genome-wide study in a cohort of Caucasian subjects demonstrated a significant association between the incidence of XFS and SNPs in the *LOXL1* gene (9). A functional complex of *LOXL1* has a prominent role in elastin fiber production (10). Down-regulation of *LOXL1* messenger RNA (mRNA), and decreased level of elastin fibers can contribute to optic nerve damage in much lower IOP in end-stage exfoliative glaucoma patients (11).

Some studies reported a marked association between several *LOXL1* gene polymorphisms, including rs1048661 and rs3825942, and susceptibility to XFS development (12, 13). This association was observed in many countries and geographic regions, including but not limited to Pakistan, Saudi Arabia, India, Australia, the United States, Austria, Germany, Italy, Finland, and Poland (1). However, rs1048661 SNP had a protective role against XFS in some reports from China, Japan and Korea (1). The prevalence of XFG is highly different between various ethnic groups (14); hence, doing case-control and genetic studies are warranted in different geographic and ethnic regions.

We aimed at evaluation of the exonic *LOXL1* SNPs, rs1048661 and rs3825942 variants in an Iranian population having XFS and XFG.

## Materials and Methods

### Subjects

Sixty subjects with XFG/XFS (44 (73.3%) males) and 40 healthy controls (18 (45.0%) males) were visited between May 2014 and May 2015 in Khatam Anbia Eye Hospital, Mashhad, Iran and included in this study. The mean ± SD age of XFG patients and control subjects were 66.0±1.7 and 70.7±1.4 yr, respectively. Detailed, informed consents were obtained from all subjects before participation in study.

The Ethics Committee of Mashhad University of Medical Sciences authorized and granted approval to the current study.

All patients and healthy controls went through an extensive ocular examination, including visual acuity testing, slit-lamp examination, Goldmann applanation tonometry, dynamic gonioscopy, evaluation of the optic disc and central corneal thickness measurement.

The diagnosis of XFS was based on the presence of exfoliative material on the anterior lens capsule, the iris tissue, or the corneal endothelium along with multiple in-office IOP measurements of less than 21 mmHg and no evidence of glaucomatous optic nerve damage. To ensure detection of any exfoliative material, the pupil was dilated in all patients and a laser peripheral iridotomy was done in that subset of patients predisposed to pupillary block. Any patient with XFS finding and coexisting high/treated IOP, glaucomatous optic nerve cupping and visual field defect, was diagnosed as XFG. Individuals older than 55 yr with no apparent exfoliative material deposition on anterior segment structures and no evidence of glaucoma or ocular hypertension were selected as healthy control subjects. All of these selected healthy individuals had IOP readings of less than 21 mmHg as well as normal optic discs.

### SNPs Genotyping

A DNA purification kit (Genomic DNA Isolation kit, GENET Bio, South Korea) was used for extraction of genomic DNA from tissue and peripheral. Primer sequences were designed by Primer Premier 5 program (Premier Biosoft International, Palo Alto, CA, USA) (Table 1). We used polymerase chain reaction (PCR) amplification to investigate the rs1048661 and rs3825942 SNPs in a 20 μl reaction volume containing 50 ng DNA, 1 pmol/μl primers, 10μl of PCR Master Mix (Amplicon, Denmark) and 7μl ddH_2_O. For all subjects, the DNA was amplified. The temperature and time for denaturation, annealing, elongation, and final extension were 95 °C for 5 min, 95 °C for 30 sec, 59 °C for 30 sec and 72 °C for 10 min, respectively. The ABI 3100 or ABI 3730 capillary sequencer (MACROGEN, South Korea) were used for sequencing.

**Table 1:** Characteristics of the primers used in this study to investigate LOXL1 mutations in exfoliative glaucoma patients

***Gene***	***Primer type***	***Primer name***	***Primer sequence***	***PCR size***	***Product accession number***
*LOXL1* (rs1048661, rs3825942)	Seq-Analysis	Forward	5′ CTTGCTCAACTCGGGCTCAGA3′	120	*NG-011466*
		Reverse	5′ TCGTAGTTCTCG-TACTGGCTGAC3′		
*LOXL1*	Real-time PCR	Forward	5′ CTGTGCTGCGAAGAAGAAGTG 3′	137	*NM_204305.1*
		Reverse	5′ AAGTCTGCTGTGCCCTGGTTC 3′		
GAPDH	Real-time PCR	Forward	5′ GAGTCCACTGGCGTCTTCAC3′	164	*NM_001289746*
		Reverse	5′ GAGGCATTGCTGATGATCTT-GAG3′		

### Real-time PCR analysis

Anterior lens capsule specimens were obtained from 15 non-glaucomatous, senile cataract patients, and 15 patients with coexisting XFG and cataract. The anterior capsulorhexis tissue was used and in combined cases, the specimen was collected before proceeding to glaucoma surgery to reduce the risk of blood contamination of the specimen. The anterior capsule obtained during cataract surgery was immediately transferred into an RNAlater reagent (Denazist, Iran) to preserve the RNA. Total RNA of the anterior lens capsules was isolated using the RNeasy mini kit plus (Qiagen, Germany). Next, cDNA was prepared using the AccuPower® RocketScript™ RT Pre-Mix, according to the manufacturer's protocol (Bioneer, South Korea). For this purpose, cDNAs were synthesized by combining 500 μg of the anterior capsules RNA, 10 pmoles Oligo dT, 10 pmoles random hexamers and 200 U reverse transcriptase enzyme in a 20 μl reaction volume. The mixture was incubated at 42 °C for 1.5 h. We used Maxima SYBR Green/ROX qPCR Master Mix Kit (Thermo, USA) to measure human *LOXL1* and *GAPDH* gene expressions. Primer Premier 5 program was used to design the primer sequences in order to amplify the target genes (Table 1). The PCR mixture (20μl total volume) was composed of 10 μl of qPCR Master Mix, 100 ng cDNA, 1 pmol/μl Primers and until the final volume ddH_2_O. The temperature and time for PCR parameters were including 95 °C for 6 min followed by 45 cycles of 95 °C for 20 sec, 65 °C for 30 sec, and 72 °C for 20 sec. Nonspecific products in the reaction were detected by melting curve analysis. No template controls (NTC) were used as negative control. RTPCR products were evaluated in 1% agarose gel. The standard curves were prepared for each gene and cDNA dilution and illustrated against respective cycle threshold (Ct) by an ABI 7300 (Applied Biosystems, Foster City, CA, USA) system. As a mean to quantify the *LOXL1* mRNA relative expression in patients' anterior lens capsule, we analyzed the qPCR outputs by the REST^©^ 2009 program (Technical University of Munich, Qiagen, Hilden, Germany) to investigate any possible difference among the groups. In our study, *GAPDH* was applied as the reference gene for expression normalization.

The evaluation of RNA integrity for anterior lens capsule tissue samples was performed using 1% agarose gel. In addition, purification of the total RNA was done by Nanodrop (Thermo Fisher Scientific, USA).

### Statistical analysis

We used FinchTV (PerkinElmer Informatics, USA) and BioEdit (Ibis Therapeutics, Carlsbad, CA, USA) programs to analyze and edit the results of sequencing (15). To estimate the Hardy-Weinberg equilibrium, correlation test, and haplotype frequency, SNPstats program (Catalan Institute Oncology, Barcelona, Spain) was used (16, 17). Association between allele and genotype frequencies and age, IOP and were investigated by SPSS (SPSS Inc. Released 2007. SPSS for Windows, ver. 16.0. Chicago, USA).

## Results

### Case-control association study

Sixty patients suffering from XFS/XFG and 40 control subjects were enrolled in this study. In our sample, CDR and age were not significantly different between those with and without XFS/XFG; however, IOP was significantly higher in the individual with XFG compared to control group (Table 2).

**Table 2:** Clinical data in case and control group

***Variable***	***Case group (n=60)***		***Control group (n=40)***		**P-*value***
	***Mean***	***SEM***	***Mean***	***SEM***	
Age(yr)	71.93	2.057	67.00	2.342	0.469
Left eye CDR	0.6800	0.26241	0.4200	0.13732	0.070
Right eye CDR	0.6800	0.18205	0.4200	0.14243	0.265
Left eye IOP	21.4000	8.11348	12.2000	2.30527	0.019*
Right eye IOP	18.6667	5.05211	11.6667	3.22195	0.032*

* Significant at the 0.05 level

The genotype distribution in both SNPs was similar to Hardy-Weinberg equilibrium. In both rs1048661 and rs3825942 SNPs, GG genotype in *LOXL1* gene exon was found to be considerably associated with an increase in the risk of XFS/XFG under recessive models (Table 3). The G allele for rs3825942 was present in all of our XFS/XFG patients (n=60, Table 3).

**Table 3:** Genotype and allele frequencies for rs3825942 and rs1048661 in patients with exfoliation syndrome and control subjects

***SNP***	***Control (n= 40)***	***XFS (n=60)***	***OR (95% CI)***	***AIC***	**P-*value***
rs1048661					
T	16	12			
G	64	108	2.250	(1.0011–5.0569)	0.0497*
Genotype					
G/T	16	12			
G/G	24	48	2.6667	(1.0900–6.5237)	0.0316*
rs3825942					
A	22	0			
G	58	120	92.6923	(5.5259–1554.8332)	0.0016*
Genotype					
A/A	1	0			
G/A	20	0			
G/G	19	60	133.41	(7.7172–2306.3195)	0.0008*

* Significant at the 0.05 level

The finding showed that A allele in rs3825942 could be considered as the main factor with utmost importance in modifying XFS/XFG. Geno-type frequencies for two polymorphisms in XFS/XFG also differed significantly in comparison to control subjects. Regarding rs1048661 SNP analysis, TT genotypes could not be seen in XFS/XFG and control populations. Moreover, GT genotypes frequency were not significantly different in XFS/XFG and control population, but we find that GG genotypes have a significant effect on XFG/XFS. The rs1048661 and rs3825942 SNPs sequencing showed the same results in both blood and anterior lens capsule specimens. We used logistic regression analysis to assess the effect of these two SNPs in patients with XFS/XFG and found that rs1048661 SNP had a significant effect on rs3825942 SNP (*P*=8.45×10^−7^). In contrast to rs1048661 SNP, rs3825942 SNP had not a significant association with XFS/ XFG (*P*=0.997). The analysis of variance in both genotypes of rs3825942 indicated a significant effect of age, CDR, and IOP in both genotypes. However, no significant differences in age, CDR and IOP were found for rs1048661 SNP between the groups (Table 4). There was no significant correlation between rs3825942 SNP and age (*P*=0.278), however; it had significant association with CDR and IOP (Table 4).

**Table 4:** SNPs data in case and control group

***Variable***	***Case and control group (n=100)***			**P-*value***
	***Locus***	***Mean***	***SEM***	
rs1048661				
Age (yr)	GG	68.73	0.969	0.486
	GT	70.18	1.864	
CDR Left	GG	0.6059	0.02496	0.333
	GT	0.5545	0.04686	
CDR Right	GG	0.556	0.0240	0.829
	GT	0.545	0.0430	
IOP Left	GG	18.4211	0.76348	0.766
	GT	17.9545	1.20511	
IOP Right	GG	16.54	0.673	0.471
	GT	15.50	1.272	
rs3825942				
Age (yr)	GG	69.53	0.945	0.278
	GA	67.24	2.008	
CDR Left	GG	0.6383	0.02421	0.000**
	GA	0.4333	0.03404	
CDR Right	GG	0.587	0.0236	0.002**
	GA	0.429	0.0339	
IOP Left	GG	19.8701	0.71655	0.000**
	GA	12.6190	0.54544	
IOP Right	GG	17.35	0.677	0.000**
	GA	12.38	0.782	

* Significant at the 0.05 level ///

** Significant at the 0.01 level

### Haplotype analysis of LOXL1 single nucleotide polymorphisms

In order to estimate the haplotype frequency, expectation-maximization algorithm was used (Table 5). The GG haplotype demonstrated the highest cumulative frequency. Between the list of the two-locus haplotypes of SNPs (rs1048661 G/T and rs3825942 G/A), the GG haplotype demonstrated an increased risk for XFG under recessive models (*P*<0.0001), compared to the GA and TG haplotypes (Table 6).

**Table 5:** Haplotype frequencies estimation (n=100)

	***rs1048661***	***rs3825942***	***Total***	***XFG***	***Control***	***Cumulative frequency***
*1*	G	G	0.7952	0.9	0.6494	0.7952
*2*	G	A	0.0948	0	0.2256	0.89
*3*	T	G	0.0948	0.1	0.0756	0.9848
*4*	T	A	0.0152	0	0.0494	1.00

**Table 6:** Haplotype association with response (n=100, crude analysis)

	***rs1048661***	***rs3825942***	***Frequency***	***OR (95% CI)***	**P-*value***
1	G	G	0.7952	4.8589	>0.0001*
2	G	A	0.0948	0.2	0.27
3	T	G	0.0948	1.2	0.84

* Significant at the 0.05 level

### RNA Extraction

**Fig. 1:**
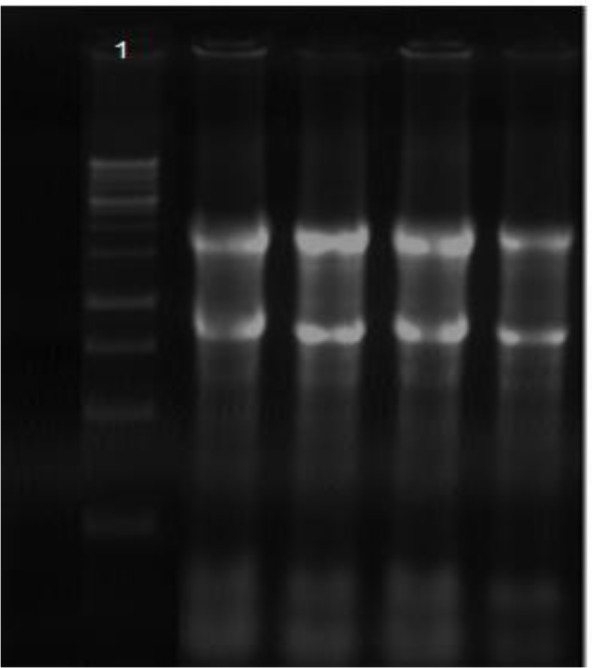
Total RNA quality assessment on the basis of 18S and 28S rRNA. Lane # 1 shows DNA ladder (1kb). The Gel Red staining pattern of intact total RNA shows clearly defined 18S and 28S ribosomal RNA bands

We evaluated RNA integrity of anterior lens capsule tissue. The samples had 2 clear bonds in 4.8 kb and 1.8 kb position which return to 28s and 18s ribosomal RNA subunits, respectively (Fig. 1).

### Quantification of LOXL1 mRNA by the use of anterior lens capsules

**Fig. 2:**
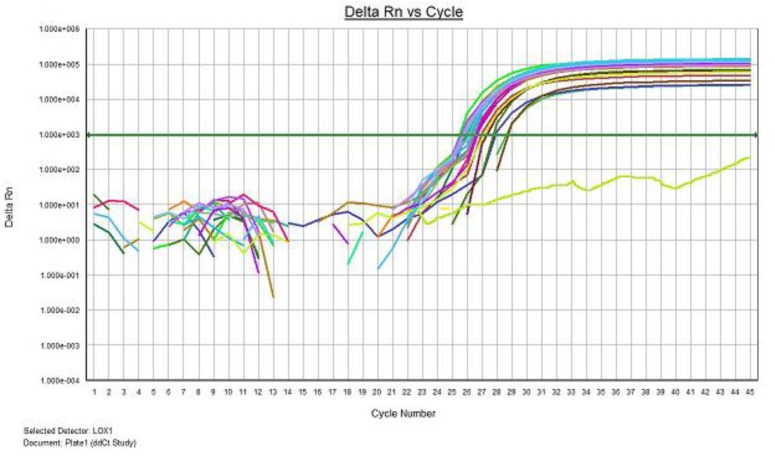
Amplification curve of LOXL1 gene expression. The amplification curve of LOXL1 are consistent and Different colors showed different samples. Negative control has not produced amplification curve

**Fig. 3:**
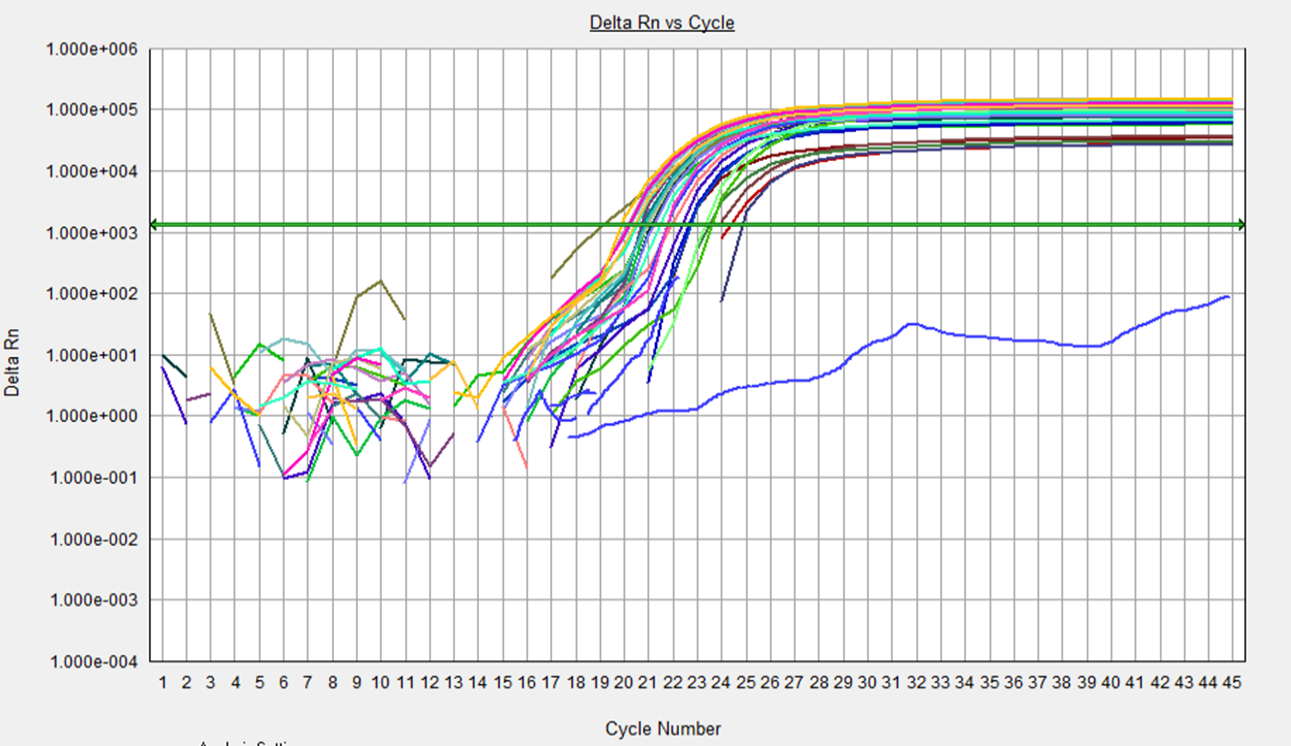
Amplification curve of GAPDH gene expression. The amplification curve of GAPDH are consistent and Different colors showed different samples. Negative control has not produced amplification curve

**Fig. 4:**
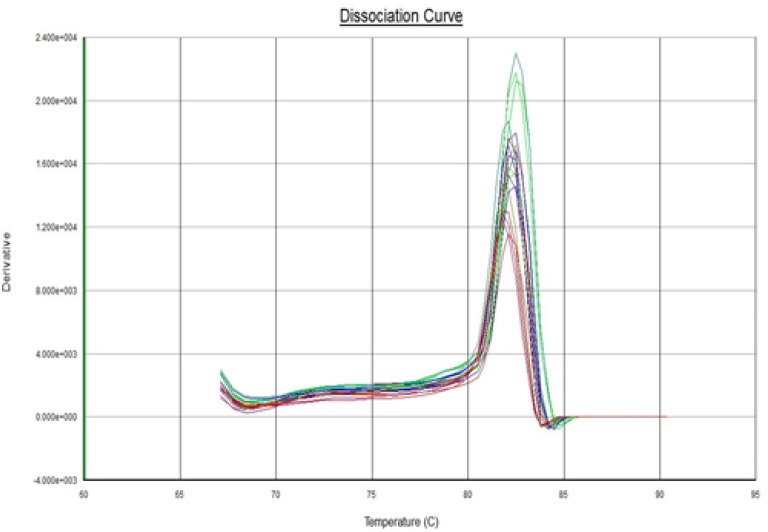
Melting curve analysis of LOXL1 gene expression. The consistent of case and control curves indicated that specificity of the Real-Time PCR reaction

**Fig. 5:**
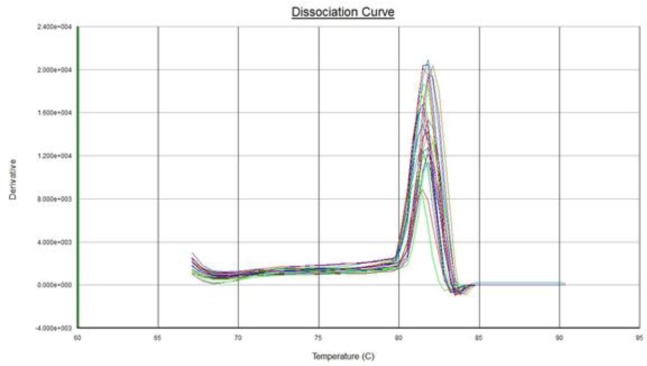
Melting curve analysis of GAPDH gene expression. The consistent of case and control curves indicated that specificity of the Real-Time PCR reaction

Real-time PCR reaction was performed for 15 XFG patients and 15 control subjects. The expression analysis demonstrated an expression of 1.824 with a standard error of 0.532–6.633 and 95% confidence interval between 0.072 and 17.900.. No significant difference was observed between the expression levels of *LOXL1* mRNA in the lens epithelium achieved from subjects with XFG and control group (*P*= 0.111) and for either SNP genotype. A normal amplification curve obtained in all samples (Figs. 2 and 3); the amplification specificity was evaluated using melting curves, which demonstrated high specificity (Figs. 4 and 5).

## Discussion

Up to now, *LOXL1* has been reported to have association with XFS and XFG in most studies. Our findings showed a significant correlation between rs1048661 and rs3825942 SNPs and XFS/XFG in Khorasan Provinces, Iran. This syndrome is less frequent in patients under 60-year-old (18, 19); hence, we only included subjects older than 60 in the current study. No significant association was observed between the age and rs1048661 and rs3825942 SNPs attributed to our inclusion criteria sampling method and the role of inheritable mutations. A wide range of cup-to-disc ratios (0.0 to 0.87) has been reported in normal population (20), and there is considerable overlap between ‘physiologic’ and ‘pathologic’ CDRs, which could probably explain the lack of correlation between the investigated SNPs and CDR (21, 22). On the other hand, IOP has been known as a fundamental risk factor in the glaucoma development and we observed a significant correlation between IOP and rs1048661 and rs3825942 SNPs in XFG patients. Numerous roles have been proposed for the lysyl oxidase proteins family, including the oxidative deamination of the lysine residues and cross-linking of elastin fibers (23). The phenotypic consequences of *LOXL1* mutations were studied in knockout mice. This gene had a crucial function in the elastic fibers homeostasis; moreover, it has an important role for normal function of trabecular meshwork (24, 25). Based on these, the *LOXL1* acts as one of the main causative genes in the pathogenesis of XFS and XFG. The rs1048661 and rs3825942 polymorphisms have been shown to be non-synonymous variants and this could affect the protein expression and function.

The importance of these SNPs in disease development is still to be elucidated, as some cohort studies demonstrated a reverse association. Our study showed a correlation of two coding *LOXL1* polymorphisms with XFS in an Iranian population for the first time. Our results appear to have considerable similarity to Caucasian populations (9, 26) and vary from Chinese (27), Japanese (13), Korean (28), and South African (29) populations.

We also noticed that in comparison to the control subjects, the G allele was more frequent in the XFS and XFG for rs3825942 and rs1048661 polymorphisms. T and A alleles have a protective role against XFS and XFG in rs1048661 and rs3825942 SNPs, respectively. Significant associations of XFG with the non-synonymous coding changes in rs1048661 and rs3825942 SNPs were reported in a Caucasian population (9). Our findings demonstrate that the effect of rs1048661 SNP on XFS is independent of rs3825942 role. Conversely, AA genotype of rs3825942 SNP is a major risk for XFS in a South African population (29). The GG genotype and the G allele are associated with the occurrence of XFS in all ethnic groups.

Recent studies have reported the expression of *LOXL1* mRNA and its protein product in various ocular tissues (29, 30); however, the effect of *LOXL1* on turn-over and formation of extracellular matrix formation has not been proved in the eye. In our study, the relative expression of *LOXL1* mRNA in the anterior lens capsules did not show a significant difference between XFG and senile cataract control subjects. In contrast to our findings, Thorleifsson et al (9) used adipose tissue and reported an upregulated TT genotype expression of *LOXL1* mRNA compared to GG and TG genotypes; the former genotype has been reported to have a protective effect against XFG in Caucasians subjects. This difference is caused as a result of the mixed *LOXL1* polymorphism genotype of elderly subjects who have cataract surgery. Because of ethical considerations, precise genotype of these subjects could not be determined. Nevertheless, the analysis of the relative expression of *LOXL1* mRNA in anterior lens capsule seems to be better than adipose tissue as it is more representative targets pathological sites (14).

## Conclusion

Polymorphisms of the *LOXL1* gene (rs3825942 and rs1048661) were associated with the prevalence of XFS/XFG in an Iranian population. To the best of our knowledge, the current study is the first research investigating the association of factors such as IOP, CDR, and age with *LOXL1* SNPs (rs3825942 and rs1048661). However, the interactions between risk factors (sex, age, environmental variables) and the *LOXL1* genes are to be clarified in future research studies. These findings can encourage future studies to investigate the association between the *LOXL1* SNPs and other possible risk factors of XFS/XFG.

## Ethical considerations

Ethical issues (Including plagiarism, informed consent, misconduct, data fabrication and/or falsification, double publication and/or submission, redundancy, etc.) have been completely observed by the authors.
